# Improving Doctors’ Knowledge and Practices on Needle-Stick Injury Prevention and Management: A Two-Cycle Clinical Audit at Al Managil Teaching Hospital

**DOI:** 10.7759/cureus.97230

**Published:** 2025-11-19

**Authors:** Ahmed Sufyan Ahmed Abdalla, Mohamed Ahmed Fathalla Idris, Ryan Osman Alhessen Saidahmed, Sanaa Mohammedelbager Mahmoud Mursy, Miska Haroun Mohamed Hassan, Shima Abdalraheem Elnadeef Hussein, Mai Fadlallah, Mohamed Murtada Hajelshafeei Mohamed, Hashim Bashir Elmadani Ahmed, Hazim Abdelhalim Osman Daoud, Hussein Abu Obaida Abdelhadi Hussein, Marwa Elaziz, Roaa Mohamed Ali Elbashir, Mogtaba Saifelyazel Halawi Yousif, Marwa Mohamed, Hind Abdelmoneim Mahjob Hajosman, Mohammed Balla Yousif Balla, Abdalmahmoud Asadig Kanan Ahmed, Waleed Abdelbasit Khider, Elwathig Abdalla

**Affiliations:** 1 General Surgery, Al Managil Teaching Hospital, Al Managil, SDN; 2 Emergency Department, Al Managil Teaching Hospital, Al Managil, SDN; 3 Obstetrics and Gynecology, Al Managil Teaching Hospital, Al Managil, SDN; 4 Plastic Surgery, Tajmeel Medical Centre, Sharjah, ARE; 5 General Surgery, Managil Teaching Hospital, Al Managil, SDN; 6 General Surgery, Nottingham University Hospitals NHS Trust, Nottingham, GBR; 7 Orthopaedics and Trauma, Al Managil Teaching Hospital, Al Managil, SDN; 8 Medicine and Surgery, Al Managil Teaching Hospital, Al Managil, SDN; 9 General Practice, Al Managil Teaching Hospital, Al Managil, SDN; 10 Medicine, Al Managil Teaching Hospital, Al Managil, SDN; 11 Trauma, Al Managil Teaching Hospital, Al Managil, SDN; 12 Orthopaedic Surgery, Al Managil Teaching Hospital, Al Managil, SDN

**Keywords:** clinical audit, needle-stick injury, occupational exposure, post-exposure prophylaxis, sharps safety

## Abstract

Background: Needle-stick injuries (NSIs) pose a major occupational hazard for doctors, serving as a route for transmission of hepatitis B virus (HBV), hepatitis C virus (HCV), and human immunodeficiency virus (HIV). International guidelines emphasize immediate wound care, avoidance of recapping, use of safety-engineered devices, and timely initiation of post-exposure prophylaxis (PEP). However, knowledge and adherence remain suboptimal in many low-resource settings. This audit evaluated doctors’ awareness, knowledge, and practices related to NSI prevention and management at Al Managil Teaching Hospital, with the aim of identifying gaps and assessing the impact of targeted interventions.

Methods: A closed-loop clinical audit was conducted in two cycles (May and September 2025) among doctors working in high-risk departments. Fifty participants were included in each cycle. Data were collected through a structured questionnaire covering demographics, sharps-safety knowledge, immediate management of NSIs, training and reporting practices, and awareness of immunization and PEP protocols. Following the first cycle, interventions included structured teaching sessions, posters, and distribution of guideline leaflets. The same questionnaire was re-administered in the second cycle to measure changes in knowledge and awareness.

Results: Substantial improvements were observed across all domains of knowledge and practice. Recognition of HBV as the most common NSI-transmitted pathogen increased from 52% to 100%. Awareness of correct first-aid measures (washing with soap and water) rose from 72% to 100%, while understanding that safety-engineered devices are the most effective preventive measure improved from 14% to 100%. Correct knowledge of sharps disposal (replacing at three-quarters full, disposal without recapping) improved from 26% and 12% at baseline to 100% in the re-audit. Training and reporting also demonstrated marked progress: formal training increased from 18% to 100%, regular refresher training from 14% to 100%, and awareness of a reporting system from 25% to 98%. Knowledge of PEP improved from 24.5% to 100% for definition and from 38% to 100% for correct initiation within 72 hours.

Conclusion: Targeted educational interventions achieved significant improvements in doctors’ knowledge and awareness of NSI prevention and management. The audit underscores the importance of recurrent training, safe sharps disposal practices, and supportive reporting systems. Sustained institutional commitment, reinforced by national guidelines and monitoring, is necessary to translate knowledge into lasting improvements in occupational safety.

## Introduction

Needlestick injuries (NSIs) are among the leading occupational hazards faced by healthcare workers and serve as a major vector for the transmission of blood-borne pathogens such as hepatitis B virus (HBV), hepatitis C virus (HCV), and human immunodeficiency virus (HIV). According to the World Health Organization (WHO), tens of thousands of new HBV and HCV infections and a substantial number of HIV cases occur annually as a result of contaminated sharp instruments, underscoring the ongoing threat these injuries pose in routine clinical practice [1].

Despite the existence of preventive guidelines, NSIs remain a persistent global concern. Evidence from systematic reviews and meta-analyses demonstrates that healthcare personnel, particularly doctors and nurses, continue to experience high exposure rates-especially in low- and middle-income countries. Contributing factors include inadequate training, inconsistent adherence to safety protocols, and limited availability of safety-engineered devices. A review conducted within the Eastern Mediterranean Region reported an overall prevalence approaching 43%, with the highest rates documented in sub-Saharan Africa [2].

The risk of NSIs varies across healthcare settings. A disproportionate number of incidents occur in high-risk areas such as emergency departments, surgical theaters, and maternity units, where staff frequently handle sharp instruments under time pressure. Unsafe practices - most notably the recapping of needles and improper disposal - further amplify vulnerability. Underreporting remains a major obstacle to prevention; data from the International Safety Center’s EPINet network show that perioperative services are among the most affected, with up to half of incidents remaining unreported, delaying timely post-exposure management [3].

International authorities, including the Occupational Safety and Health Administration (OSHA) and the U.S. Centers for Disease Control and Prevention (CDC), have established clear preventive measures. These include universal HBV vaccination, the elimination of needle recapping, structured exposure-control plans, use of safety-engineered devices, and comprehensive training programs on post-exposure management [4].

However, persistent challenges continue to affect healthcare facilities in the Eastern Mediterranean and African regions. Limited access to safety devices, inadequate training coverage, inconsistent reporting systems, and low HBV vaccination rates all contribute to ongoing risk. Recent studies from Sudan further confirm these issues, revealing high exposure levels, frequent recapping practices, and considerable underreporting.

In this context, a quality-improvement audit was conducted at Al Managil Teaching Hospital to evaluate doctors’ knowledge and practices regarding NSI prevention and post-exposure management. The project aimed to identify gaps in awareness and reporting behavior, assess compliance with institutional safety standards, and implement targeted educational interventions to enhance sharps safety and reduce preventable occupational exposures [5].

## Materials and methods

Study design and setting

This closed-loop clinical audit was conducted at Al Managil Teaching Hospital, a tertiary-level facility providing a wide range of medical services, including surgery, internal medicine, pediatrics, obstetrics and gynecology, and emergency medicine. High procedural volumes and the frequent use of sharp instruments make these departments particularly prone to occupational injuries.

Following the classical audit paradigm, the project was structured into four phases: baseline survey (Cycle 1), gap analysis (Cycle 2), targeted intervention implementation (Cycle 3), and re-audit (Cycle 4) to evaluate outcomes [6]. The first cycle was performed in May 2025 over two weeks, followed by an intervention period from mid-May to June 2025. The second cycle concluded in September 2025. Rather than quantifying the absolute number of injuries, the audit primarily sought to assess doctors’ knowledge of sharps-safety protocols and their adherence to post-exposure management practices.

Audit standards

Evaluation criteria were derived from internationally recognized frameworks, principally the OSHA Bloodborne Pathogens Standard, which mandates engineering and work-practice controls such as the use of safety-engineered devices, prohibition of two-handed recapping, availability of sharps containers at the point of use, hepatitis B vaccination, and post-exposure evaluation [7].

These principles were reinforced by the WHO Best Practices for Injections and Related Procedures Toolkit, emphasizing universal precautions, proper sharps disposal, and elimination of recapping [8]. Initial management procedures were guided by recommendations from the CDC, National Institute for Occupational Safety and Health (NIOSH), and the U.S. Public Health Service (USPHS), which stress prompt wound irrigation, early medical evaluation, and initiation of post-exposure prophylaxis (PEP) - ideally within hours and no later than 72 hours [9,10].

Additional standards included baseline and serial serological testing, HIV prophylaxis when indicated, and monitoring for HCV infection [9,10]. The Advisory Committee on Immunization Practices (ACIP) and CDC guidelines informed hepatitis B vaccination protocols, including post-vaccination antibody testing and immunization of all at-risk employees [11].

Based on these international frameworks, measurable local audit goals were established to guide evaluation and improvement. The audit sought to ensure that every doctor had attended at least one sharps-safety training session within the previous year and demonstrated universal awareness of the no-recap policy. In addition, it aimed for at least 95% of doctors to correctly describe the appropriate actions following a needlestick injury, including immediate first aid, timely reporting, and initiation of post-exposure management. Comparable awareness levels were also expected regarding hepatitis B vaccination, HIV post-exposure prophylaxis, and follow-up testing protocols.

Study population

The study population included all physicians working in high-risk departments of Al Managil Teaching Hospital. Because they perform a greater number of invasive procedures and have less clinical experience, junior doctors - particularly house officers and medical officers - represented the majority of participants. Specialists from surgery, internal medicine, pediatrics, obstetrics and gynecology, and emergency medicine were also included.

A matched sample of 50 doctors was recruited for each audit cycle (pre- and post-intervention). This number reflected the total number of eligible doctors available during the data collection periods and allowed proportional representation from each major department. Inclusion criteria were at least three months of hospital experience and active clinical duties during the study period; physicians absent for more than three weeks of leave were excluded.

Data collection

To collect data, the audit team worked with the infection control unit to create a systematic, self-administered questionnaire. The questionnaire was proofread and piloted to make sure it was easy to understand and reliable before it was put into use, following guidelines set out by OSHA, WHO, and the CDC [7,8,10]. The survey inquired about participants' familiarity with sharps safety measures, such as the no-recap policy and the proper usage of sharps containers, as well as their demographic information, such as clinical department and years since graduation. Additionally, it tested participants' knowledge of what to do in the event of a needlestick injury, with an emphasis on first aid and reporting protocols. Sections that followed tested comprehension of hepatitis B vaccination, HIV prophylaxis, suggested follow-up testing, and familiarity with institutional reporting procedures, as well as attendance at prior sharps-safety training sessions.

Both audit cycles used the same questionnaire. Targeted interventions were implemented in response to gaps in awareness and practice identified in the May 2025 baseline survey. In September 2025, after implementation, the questionnaire was re-administered to assess improvement. All 50 distributed questionnaires per cycle were returned and included in the analysis. Some participants left one or two items unanswered; however, all percentages were based on n = 50. Consequently, absolute counts for certain response options may be fewer than 50, but no questionnaires were excluded

Interventions between cycles

Targeted interventions were designed to address deficiencies identified in the baseline evaluation. Infection-control officers conducted interactive workshops covering proper post-exposure management, compliance with the no-recap policy, and safe handling of sharps.

Educational posters placed in clinical departments reinforced emergency procedures, reporting channels, and PEP availability. Departmental meetings further emphasized adherence to safe practices and promoted a culture of supportive, non-punitive reporting. To ensure ongoing access to reference materials, printed guideline leaflets summarizing institutional protocols for needlestick-injury management were distributed at points of care. All facilitators underwent brief orientation sessions to standardize workshop content and delivery across departments.

Data analysis

Responses were entered into Microsoft Excel (Redmond, WA, USA) and double-checked for accuracy before analysis. Descriptive statistics (frequencies and percentages) summarized awareness and compliance levels. Compliance with standards was calculated as the proportion of correct responses.

Comparisons between the first and second cycles were performed using the chi-square test to determine statistical significance, with a p-value < 0.05 considered significant. Tables and graphs were generated to illustrate baseline deficiencies, post-intervention improvements, and remaining gaps requiring reinforcement.

Ethical considerations

Ethical approval for this audit was obtained from the Institutional Review Board (IRB) of Al Managil Teaching Hospital, Gezira State, Ministry of Health (IRB number QIP-2025-00867). The approval confirmed that the audit met the ethical and methodological standards required for implementation under the hospital’s Quality Improvement (QI) program. Participation was entirely voluntary, and completion of the questionnaire was considered implied consent. All responses were anonymized, and no personal identifiers were collected to maintain confidentiality. As the project focused on healthcare staff knowledge and practices without patient involvement, it was classified as a QI initiative rather than human subjects’ research.

## Results

A total of 50 doctors participated in each audit cycle. The professional composition demonstrated a clear shift following the intervention. In the first cycle, half of the respondents (25, 50%) classified themselves under “other” positions, while house officers represented 15 (30%) and medical officers eight (16%). By the second cycle, the distribution became more balanced, with medical officers increasing markedly to 19 (38%) and registrars to five (10%), whereas the proportion of “other” positions declined to eight (16%).

Regarding year of graduation, Cycle 1 included a wide range of cohorts from 2018 to 2025. The largest groups were graduates of 2024 (eight, 16%) and 2025 (six, 12%). In contrast, Cycle 2 was dominated by 2023 graduates (20, 40%), followed by those from 2021 (eight, 16%), 2024 (eight, 16%), and a combined “other years” category (13, 26%).

Departmental representation also showed notable shifts. In Cycle 1, participation was highest from “other” specialties (15, 30%), followed by general surgery (10, 20%) and general medicine (nine, 18%). Pediatrics and emergency medicine accounted for five (10%) and eight (16%), respectively. In Cycle 2, general surgery (14, 28%) and pediatrics (13, 26%) became the most represented departments, while “others” decreased to nine (18%).

Overall, the re-audit sample reflected a larger proportion of junior doctors, recent graduates, and participants from core procedural specialties, aligning with the audit’s focus on occupational safety practices among frontline staff (Table 1).

**Table 1:** Demographic Characteristics of Doctors Participating in the Audit Across Both Cycles (n = 50 per cycle) Values are presented as frequency with percentage in brackets. Total participants per cycle = 50. Although a few respondents left one or two items unanswered, all percentages were calculated using the total sample size (n = 50) for consistency across the dataset. “Other years” in Cycle 2 includes graduates from years outside 2021–2024. “Others” in department distribution represents doctors from specialties not specified individually.

Item	Cycle 1 (n = 50)	Cycle 2 (n = 50)	Change / Trend
Current Position – House Officer	15 (30%)	17 (34%)	Slight increase
Current Position – Medical Officer	8 (16%)	19 (38%)	Marked increase
Current Position – Registrar	2 (4%)	5 (10%)	Increased
Current Position – Consultant	0 (0%)	0 (0%)	No change
Current Position – Other	25 (50%)	8 (16%)	Major decrease
Year of Graduation – 2018	2 (7%)	–	Not represented in Cycle 2
Year of Graduation – 2020	2 (7%)	–	Not represented in Cycle 2
Year of Graduation – 2021	3 (6%)	8 (16%)	Increased
Year of Graduation – 2022	4 (8%)	–	Not represented in Cycle 2
Year of Graduation – 2023	5 (10%)	20 (40%)	More than doubled
Year of Graduation – 2024	8 (16%)	8 (16%)	No change
Year of Graduation – 2025	6 (12%)	–	Not represented in Cycle 2
Year of Graduation – Other Years	–	13 (26%)	New category in Cycle 2
Department – General Surgery	10 (20%)	14 (28%)	Increase
Department – Obstetrics & Gynecology	3 (6%)	4 (8%)	Slight increase
Department – General Medicine	9 (18%)	8 (16%)	Slight decrease
Department – Pediatrics	5 (10%)	13 (26%)	Large increase
Department – Emergency Medicine	8 (16%)	–	Not separately listed in Cycle 2
Department – Others	15 (30%)	9 (18%)	Decrease

Knowledge regarding blood-borne pathogens and sharps safety showed substantial improvement between the two audit cycles. In the first cycle, just over half of the doctors (26, 52%) correctly identified HBV as the most common pathogen transmitted through needlestick injuries, while a considerable proportion incorrectly selected HIV (17, 34%) or HCV (four, 8%), and three respondents (6%) were unsure. By the second cycle, all participants (50, 100%) correctly identified HBV, eliminating misconceptions and uncertainty.

Awareness of immediate first-aid measures also improved markedly. Initially, 36 doctors (72%) recognized washing the wound with soap and water as the correct first step, while nine (18%) incorrectly reported sucking the wound and five (10%) expressed uncertainty. In the re-audit, all doctors (50, 100%) provided the correct response, indicating universal awareness of the recommended protocol.

Preventive knowledge regarding safe practices demonstrated the most striking changes. In the baseline survey, only seven doctors (14%) identified safety-engineered devices as the most effective means to reduce risk, whereas the majority (33, 66%) incorrectly believed that recapping needles was appropriate. By the second cycle, all respondents (50, 100%) selected safety-engineered devices, reflecting a complete shift toward correct knowledge.

Similar improvements were observed in sharps-disposal practices. In Cycle 1, only 13 doctors (26%) correctly stated that sharps boxes should be replaced when three-quarters full, compared with universal awareness in Cycle 2 (50, 100%). Likewise, safe disposal without recapping increased from six (12%) to 50 (100%), while the unsafe practice of recapping prior to disposal dropped from 40 (80%) to zero.

Overall, the re-audit demonstrated near-universal correct knowledge and alignment with recommended standards for pathogen recognition, immediate management, and preventive practices, confirming the effectiveness of the educational interventions (Table 2).

**Table 2:** Knowledge of Needlestick Injuries and Prevention Among Doctors in Both Audit Cycles (n = 50 per cycle) Values are expressed as frequency (percentage). Total participants per cycle = 50. Although a few respondents left one or two items unanswered, all percentages were calculated using the total sample size (n = 50) for consistency across the dataset. The “Comment” column indicates the correct answers as defined by international standards (OSHA, WHO, CDC) and highlights whether knowledge or practices improved accordingly. NSI = Needlestick Injury; HBV = Hepatitis B Virus; HCV = Hepatitis C Virus; HIV = Human Immunodeficiency Virus; OSHA = Occupational Safety and Health Administration; WHO = World Health Organization; CDC = Centers for Disease Control and Prevention.

Item	Cycle 1 (n=50)	Cycle 2 (n=50)	Comment (Correct Answer)
Most common pathogen transmitted through NSI – HBV	26 (52%)	50 (100%)	Correct answer: HBV – improved from half to universal recognition
Most common pathogen – HIV (incorrect)	17 (34%)	0 (0%)	Incorrect option – dropped to zero
Most common pathogen – HCV (incorrect)	4 (8%)	0 (0%)	Incorrect option – eliminated
Most common pathogen – Don’t know	3 (6%)	0 (0%)	Uncertainty resolved
First step after NSI – Wash with soap and water	36 (72%)	50 (100%)	Correct answer – universal awareness by Cycle 2
First step – Suck wound (incorrect)	9 (18%)	0 (0%)	Unsafe practice eliminated
First step – Don’t know	5 (10%)	0 (0%)	Uncertainty eliminated
Most effective prevention – Safety-engineered devices	7 (14%)	50 (100%)	Correct answer – major improvement in knowledge
Most effective prevention – Recapping (incorrect)	33 (66%)	0 (0%)	Unsafe misconception removed
When to stop using sharps box – 3/4 full	13 (26%)	50 (100%)	Correct answer – full compliance achieved
Usual method of disposal – Direct disposal without recapping	6 (12%)	50 (100%)	Correct answer – safe practice universally adopted
Usual method of disposal – Recap before disposal (incorrect)	40 (80%)	0 (0%)	Unsafe practice eliminated

Training and reporting practices demonstrated major improvements across the audit cycles. In Cycle 1, fewer than one in five doctors (nine, 18%) reported having received formal training on needlestick injury prevention, compared with universal training in Cycle 2 (50, 100%). Participation in refresher training also showed a substantial shift. At baseline, only seven doctors (14%) attended regular refresher sessions, while 20 (40%) participated occasionally and 23 (46%) had never attended. Following the intervention, all respondents (50, 100%) reported regular refresher training, reflecting the successful establishment of a structured and continuous education program.

Self-reported confidence in handling NSIs improved correspondingly. In Cycle 1, only 11 doctors (22%) felt adequately trained, whereas in Cycle 2, this increased to all respondents (50, 100%). Awareness of a formal reporting system also strengthened markedly, rising from 13 doctors (26%) in Cycle 1 to 49 (98%) in Cycle 2.

Finally, when asked what measures could further reduce the risk of NSIs, the overwhelming majority of respondents in both cycles emphasized the need for continued training and education. This view, expressed by 47 doctors (94%) in Cycle 1 and all participants (50, 100%) in Cycle 2, underscores a strong and consistent recognition of the central role of continuous professional development in promoting occupational safety (Table 3).

**Table 3:** Training and Reporting on Needlestick Injuries Among Doctors Across Both Audit Cycles (n = 50 per cycle) Values are presented as frequency (percentage). Total participants per cycle = 50. Although a few respondents left one or two items unanswered, all percentages were calculated using the total sample size (n = 50) for consistency across the dataset. Comments column indicates the correct standard based on OSHA/WHO/CDC recommendations. NSI = Needlestick Injury; OSHA = Occupational Safety and Health Administration; WHO = World Health Organization; CDC = Centers for Disease Control and Prevention.

Item	Cycle 1 (n = 50)	Cycle 2 (n = 50)	Comment (Correct Answer)
Received formal training on NSI prevention	9 (18%)	50 (100%)	Correct answer: all staff should receive formal training – achieved in Cycle 2
Participation in refresher training – Regularly	7 (14%)	50 (100%)	Correct answer: refresher training should be regular – achieved in Cycle 2
Participation in refresher training – Occasionally	20 (40%)	0 (0%)	Occasional training not sufficient – eliminated by Cycle 2
Participation in refresher training – Never	23 (46%)	0 (0%)	No participation – eliminated by Cycle 2
Felt adequately trained to handle NSIs	11 (22%)	50 (100%)	Correct answer: staff should feel adequately trained – achieved in Cycle 2
Presence of a formal reporting system	13 (26%)	49 (98%)*	Correct answer: workplace should have a formal reporting system – nearly universal in Cycle 2
Believed more training and education needed	47 (94%)	50 (100%)	Consistent with international standards; reflects culture of continuous improvement

Knowledge of PEP protocols improved dramatically between the two audit cycles (Table 4). In the first cycle, only 12 doctors (24%) correctly defined PEP as medication given to prevent infection after exposure, while nearly half (24, 48%) mistakenly believed it referred to a vaccine administered before exposure. Smaller proportions selected blood test after injury (nine, 18%) or were uncertain (four, 8%). By the second cycle, all participants (50, 100%) provided the correct definition, demonstrating universal understanding of this essential safety protocol.

**Table 4:** Knowledge of Post-Exposure Prophylaxis (PEP) Among Doctors Across Both Audit Cycles (n = 50 per cycle) Values are presented as frequency (percentage). Total participants per cycle = 50. Although a few respondents left one or two items unanswered, all percentages were calculated using the total sample size (n = 50) for consistency across the dataset. Comments column indicates the correct standard based on CDC/USPHS guidelines for occupational HIV exposure. PEP = Post-Exposure Prophylaxis; CDC = Centers for Disease Control and Prevention; USPHS = United States Public Health Service; HIV = Human Immunodeficiency Virus.

Item	Cycle 1 (n = 50)	Cycle 2 (n = 50)	Comment (Correct Answer)
Definition of PEP – Medication given to prevent infection after exposure	12 (24%)	50 (100%)	Correct answer – universal recognition by Cycle 2
Definition of PEP – Vaccine before exposure (incorrect)	24 (48%)	0 (0%)	Incorrect option – eliminated
Definition of PEP – Blood test after injury (incorrect)	9 (18%)	0 (0%)	Incorrect option – eliminated
Definition of PEP – Don’t know	4 (8%)	0 (0%)	Uncertainty eliminated
Recommended initiation of PEP – Within 72 hours	19 (38%)	50 (100%)	Correct answer – universal recognition by Cycle 2
Recommended initiation of PEP – Within 24 hours (partially correct)	16 (32%)	0 (0%)	Misconception removed
Recommended initiation of PEP – Within 1 hour (overestimation)	5 (10%)	0 (0%)	Misconception removed
Recommended initiation of PEP – Within 1 week (incorrect)	4 (8%)	0 (0%)	Incorrect option – eliminated
Recommended initiation of PEP – Don’t know	6 (12%)	0 (0%)	Uncertainty eliminated

Similarly, clarity regarding the timing of PEP initiation strengthened substantially. In Cycle 1, 19 doctors (38%) correctly identified the recommended window of within 72 hours, whereas others selected shorter but inaccurate timeframes (16 (32%) within 24 hours; five (10%) within one hour), longer unsafe delays (four (8%) within one week), or were unsure (six, 12%). In Cycle 2, all respondents (50, 100%) accurately reported the correct timeframe of within 72 hours.

These findings highlight the effectiveness of the educational intervention in addressing initial knowledge gaps and achieving universal awareness of both the definition and urgency of PEP among healthcare staff (Table 4).

## Discussion

Following the targeted educational interventions, participating doctors demonstrated a significant improvement in their knowledge of NSI prevention and management. The most notable gains were observed in the ability to identify HBV as the most common pathogen transmitted through NSIs, the understanding of PEP and its appropriate timing, and the correct procedures for safe sharps disposal.

These improvements are consistent with international safety frameworks. Quick wound irrigation, avoidance of needle recapping, timely initiation of PEP, and adherence to HBV vaccination policies are key recommendations outlined in the WHO Injection Safety Guidelines, the OSHA Bloodborne Pathogens Standard, and the CDC/USPHS protocols [7,8,10]. The findings of this audit suggest that structured educational programs can significantly enhance compliance with these standards. However, sustaining such progress requires continued institutional support and reinforcement at multiple organizational levels.

Comparable findings have been reported in various settings. Systematic reviews have shown that training and the consistent application of safety measures substantially reduce the risk of NSIs, though global prevalence remains high [12]. In Abha City, studies revealed frequent underreporting and persistent misconceptions about recapping despite awareness of the correct procedures [13]. Similarly, audits from Karachi’s public tertiary hospitals documented high rates of NSIs and inadequate preventive measures [14]. In line with our observations, these reports emphasize the importance of ongoing education, accessible reporting mechanisms, and reinforcement of safe workplace behaviors.

The marked improvement in knowledge observed during this audit likely resulted from the focus placed on these key elements through workshops, posters, and departmental discussions. Conversely, persistent obstacles - including high staff turnover, competing workload demands, and limited trust in non-punitive reporting systems - may explain why awareness of reporting mechanisms and training coverage remained suboptimal during the baseline cycle. This interpretation aligns with global research demonstrating that NSIs are often underreported due to concerns about blame, administrative burden, and skepticism regarding the usefulness of follow-up procedures [15].

The predominance of junior doctors among those reporting NSIs in this audit likely reflects their greater procedural exposure and limited practical experience compared with senior colleagues. Early-career physicians frequently perform a higher volume of invasive procedures - such as cannulations, suturing, and venipuncture - often under time pressure and in high-demand environments. These circumstances increase their vulnerability to accidental injuries. This highlights the need for close supervision, skill-based mentorship, and structured hands-on training early in clinical practice to foster lasting adherence to sharps-safety protocols.

The mixed outcomes observed in this audit mirror global patterns. In low- and middle-income countries, where safety-engineered devices are scarce and underreporting remains prevalent, NSIs continue to represent a major occupational hazard [12]. Studies from sub-Saharan Africa show that only a minority of affected clinicians report incidents or receive appropriate follow-up care [16]. These parallels indicate that the challenges identified in our setting are reflective of broader systemic shortcomings rather than isolated institutional failings.

The findings reinforce the need for robust occupational health programs that extend beyond knowledge acquisition. Sustainable improvements require organizational commitment to refresher training, reliable supply of safety-engineered devices, and the establishment of supportive, non-punitive reporting mechanisms. Regular monitoring and policy reinforcement are essential to ensure that improved awareness translates into safer practices.

This audit’s major strengths include its closed-loop design, which enabled objective measurement of improvement across two cycles, and its focus on physicians - a high-risk professional group with frequent exposure to sharps. The use of structured interventions (educational workshops, posters, and guideline leaflets) allowed for targeted correction of identified gaps, and the inclusion of multiple high-risk departments enhanced the relevance of findings for frontline practice.

Nevertheless, certain limitations must be acknowledged. The single-center design restricts generalizability to other institutions, and the modest sample size, determined by the number of available doctors, may limit statistical power. Reliance on self-reported questionnaires introduces the possibility of recall or social-desirability bias. Furthermore, staff turnover and workload variation could have influenced the consistency of participation between audit cycles. The sustainability of improvements also depends on continued institutional commitment, refresher training, and integration of sharps-safety education into staff induction programs.

Future efforts should focus on embedding NSI prevention into mandatory induction and annual refresher courses, fostering open and supportive reporting cultures, and collaborating with national health authorities to develop standardized monitoring and surveillance systems. Overall, this audit demonstrates that targeted educational interventions can substantially improve doctors’ knowledge and practice regarding NSI prevention and management; however, long-term progress requires sustained institutional engagement and system-level reform.

## Conclusions

This audit demonstrated that targeted educational interventions led to a marked improvement in doctors’ knowledge and awareness of NSI prevention and management. Significant gains were observed in the recognition of HBV as the most common pathogen transmitted via NSIs, appropriate first-aid responses, safe sharps disposal, and timely initiation of post-exposure prophylaxis. These outcomes reflect measurable enhancement in understanding and self-reported adherence to safety protocols following structured educational efforts.

However, as the data were derived from self-reported questionnaires, the findings primarily reflect perceived rather than directly observed behavioral change. The results underscore the importance of sustaining progress through continuous refresher training, reliable access to safety-engineered devices, and reinforcement of supportive, non-punitive reporting systems. Long-term institutional commitment - complemented by national policies, standardized surveillance mechanisms, and ongoing monitoring - is essential to translate improved knowledge into consistent clinical practice and to minimize the risk of preventable occupational exposures among healthcare workers.

## Appendices

Instructions: This questionnaire was used to assess doctors’ knowledge, attitudes, and practices regarding needlestick injury (NSI) prevention and management. It was distributed in both audit cycles and completed anonymously. Please select the most appropriate answer for each question.

Section A: Demographic Information

Current Position: ☐ House Officer ☐ Medical Officer ☐ Registrar ☐ Consultant ☐ Other (please specify): ____________

Year of Graduation: ☐ 2018 ☐ 2019 ☐ 2020 ☐ 2021 ☐ 2022 ☐ 2023 ☐ 2024 ☐ 2025 ☐ Other: ____________

Department: ☐ General Medicine ☐ General Surgery ☐ Pediatrics ☐ Obstetrics & Gynaecology ☐ Emergency Medicine ☐ Other: ____________

Section B: Knowledge and Awareness

Which pathogen is most commonly transmitted through needlestick injury (NSI)? ☐ Hepatitis B virus (HBV) ☐ Hepatitis C virus (HCV) ☐ Human Immunodeficiency Virus (HIV) ☐ Don’t know

What is the first action to take after sustaining a needlestick injury? ☐ Wash the area with soap and water ☐ Suck the wound ☐ Apply antiseptic directly ☐ Don’t know

Which of the following is the most effective preventive measure against needlestick injuries? ☐ Recapping needles after use ☐ Using safety-engineered devices ☐ Wearing double gloves ☐ Don’t know

When should a sharps container be replaced? ☐ When half full ☐ When three-quarters full ☐ Only when completely full ☐ Don’t know

How do you usually dispose of used needles? ☐ Recap before disposal ☐ Dispose directly without recapping ☐ Leave in tray for others to discard ☐ Don’t know

Section C: Training and Practice

Have you ever received formal training on needlestick injury prevention? ☐ Yes ☐ No

How often do you attend refresher or update training on NSI prevention? ☐ Regularly ☐ Occasionally ☐ Never

Do you feel adequately trained to manage a needlestick injury if it occurs? ☐ Yes ☐ No ☐ Not sure

Does your workplace have a formal reporting system for occupational injuries (e.g., NSI reporting form or hotline)? ☐ Yes ☐ No ☐ Not sure

In your opinion, what could further reduce the risk of needlestick injuries? ☐ More training and education ☐ Better availability of safety devices ☐ Improved supervision and feedback ☐ Other (please specify): ____________

Section D: Post-Exposure Prophylaxis (PEP)

What does the term “Post-Exposure Prophylaxis (PEP)” mean? ☐ Medication given to prevent infection after exposure ☐ Vaccine administered before exposure ☐ Blood test after injury ☐ Don’t know

Within what time period should PEP ideally be initiated after an exposure? ☐ Within 1 hour ☐ Within 24 hours ☐ Within 72 hours ☐ Within 1 week ☐ Don’t know

End of Questionnaire

This tool was adapted from international occupational safety guidelines (WHO, CDC, and OSHA) and used to assess baseline and post-intervention knowledge, attitude, and practice among doctors in the audit [7,8,10].
